# Supplementary material for: Challenges and Future of Drug-Induced Liver Injury Research—Laboratory Tests
Source: Int J Mol Sci. 2022 May 27;23(11):6049. doi: 10.3390/ijms23116049 (PMC9181520; doi:10.3390/ijms23116049)
Supplement: Supplementary file 1 [file ijms-23-06049-s001.zip › ijms-1718924-supplementary.pdf]

Suppl. Table S1 - RECAM – Revised electronic causality assessment method

	Assessment
**Domain 1 a & 1b – Replaced Item 1 (**No differentiation between hepatocellular and cholestatic type nor between initial and subsequent treatment)	
◦ **Onset after drug start (days after drug start, with day 1 being the first day the drug was taken)**	
• ≤ 1 day	-6 *
• 2 – 9 days	3
• 10 – 60 days	4
• 61 – 90 days	2
• > 90 days	0
◦ **Onset after drug stop (days after drug stop, with day 1 being the first day the drug was not taken) [for long half-life agents [2] enter zero points for Domain 1b]**	
• ≤ 30 days	0
• 31 – 60 days	-1
• 61 – 90 days	-2
• 91 – 120 days	-4
• > 120 days	-6 *
**Domain 2: Dechallenge or wash-out – Replaced item 2**	
Initial R value [1] > 5: Use ALT for washout criteria	
Initial R value [1] ≤ 5: Use ALP or TBIL for washout criteria, whichever gives a higher score	
o **Decline of ALT, ALP or TBIL to < 50 % of peak**	
• > 50 % decline occurs with continuation of drug	-6 *
• Days from peak value to < 50 % of peak	
- 1 – 30 days	4
- 31 – 90 days	3
- 91 – 182 days	2
- 183 – 365 days	1
- > 365	0

• All other instances with either no decline of ALT, ALP or TBIL or decline of < 50 % of peak	0
• ALT, ALP or TBIL is > 90 % of peak at any time > 182 days and prior or any transplant without other explanation or persistent elevation	-6 *
**Domain 3: Literature supporting liver injury – Replaced item 6**	
LiverTox Category [3] (reference: https://www.ncbi.nlm.nih.gov/books/NBK547852/)	
• LiverTox Category A or B	3
• LiverTox Category C, D or E*	1
• E or X	0
**Domain 4: Exclusion of competing causes of liver injury – Replaced item 5**	
o **Hepatitis A**	
• Missing IgM anti-HAV data	-3
• IgM anti-HAV negative (if total anti-HAV is negative, consider IgM negative as well	0
• IgM anti-HAV positive	-6 *
o **Hepatitis B**	
• Missing IgM anti-Hbc	-3
• HbsAg and IgM anti-Hbc negative	0
• HbsAg positive and IgM anti-Hbc negative	-1
• IgM anti-Hbc positive regardless of HbsAg result or missing HbsAg result	-6 *
o **Hepatitis C**	
• Missing anti-HCV or HCV RNA	-3
• Anti-HCV and HCV RNA both negative	0
• Anti-HCV and/or HCV RNA positive, score according to initial R-value [1]:	
o R ≤ 5 HCV-RNA negative and anti-HCV positive	0
o R ≤ 5 HCV-RNA positive and anti-HCV positive or HCV-RNA positive and anti-HCV negative	-1
o R > 5 with known chronic HCV infection	-1
o R > 5, no known chronic infection and no exposure risk in the last ≤ 100 days prior to onset	-1
o R > 5, no known chronic infection and no exposure risk in the last ≤ 100 days prior to onset	-6 *

○ **Hepatitis E**	
• Missing IgM anti-HEV data	-3
• IgM anti-HEV negative	0
• IgM anti-HEV positive	-6 *
○ **Alcohol (use AST and ALT values at onset)**	
• AST/ALT ≥ 2 with AST ≤ 500 and missing history of excessive alcohol intake	-3
• AST/ALT < 2 and/or AST > 500	0
• AST/ALT ≥ 2 with AST ≤ 500 and history of excessive alcohol intake, then score as below:	
○ Average of ≤ 2 SD/d for women, ≤ 3 SD/d for men within 6 weeks of onset	0
○ Average of 3-4 SD/d for women, 4-6 SD/d for men within 6 weeks of onset	-3
○ Average of ≥ 5 SD/d for women, ≥ 7 SD/d for men within 6 weeks of onset	-6 *
○ **Biliary or parenchymal disease (assessed by imaging)**	
• Missing imaging data	-3
• Imaging shows no biliary stenosis or obstruction and no or < 50 % malignant infiltration	0
• Imaging shows biliary stenosis or obstruction or ≥ 50 % malignant infiltration of the liver	-6 *
○ **Autoimmune hepatitis**	
**a) Assessment for non-minocycline and non-nitrofurantoin cases**	
• Missing ANA, ASMA and IgG	-3
• ANA < 1:80, ASMA < 1:80, IgG < 1.1 ULN (1-2 can be missing of these, those others must be below given levels)	0
• ANA ≥ 1:80 or ASMA ≥ 1:80 or IgG ≥ 1.1 x ULN	-1
• ANA ≥ 1:80 or ASMA ≥ 1:80 and IgG ≥ 1.1 x ULN and liver biopsy with typical AIH features	-6 *
**b) Assessment for non-minocycline and non-nitrofurantoin cases**	
• Missing ANA, ASMA and IgG	-3
• ANA < 1:80, ASMA < 1:80, IgG < 1.1 ULN (1-2 can be missing of these, those others must be below given levels)	0
• ANA ≥ 1:80 or ASMA ≥ 1:80 or IgG ≥ 1.1 x ULN	-1
○ **Liver injury due to ischemic liver injury (shock liver) and/pr acute congestive hepatopathy**	-3
• No information on hypoxia, hypotension shock or acute congestive hepatopathy (history incomplete or inadequate)	0

• No known or suspected prolonged hypoxia, hypotension shock or acute congestive hepatopathy within one week of onset	-6 *
• Known or suspected prolonged hypoxia, hypotension shock or acute congestive hepatopathy within one week of onset	
○ **Sepsis causing cholangitis**	
• No information on sepsis or SIRS and R-value [1] < 5	-3
• R-value [1] ≤ 5 but no sepsis or SIRS or R-value [1] > 5	0
• Sepsis or SIRS and R-value [1] < 5	-6 *
• Reaction labeled in the product characteristics	+2
• Reaction published but unlabelled	+1
• Reaction unknown	0
**Domain 5: Additional Data – Items were added when compared to RUCAM (the following information may be available but is not required)**	
**Retrospective rechallenge**	
• No history of prior exposure or no DILI with jaundice after exposure to the same agent in the past	0
• Positive history of DILI with jaundice after exposure to the same agent in the past, no documentation by lab results necessary	1
**Prospective rechallenge**	
• No rechallenge or no data regarding rechallenge	0
• Re-exposure results in rise in liver enzymes 2 – 3 x ULN (or baseline)	0
• Re-exposure: same R-value category, latency < 60 days, ALT/AST > 3 x ULN (or baseline) or ALP > 2 x ULN (or baseline)	6
• Re-exposure to the same agent results in no injury or < 2 x ULN rise in liver enzymes	-3
**Liver biopsy**	
• Biopsy not done	0
• Non-diagnostic (can be suggestive of DILI but not diagnostic)	0
• Biopsy carries features consistent with a specific DILI	1
• Diagnostic of non-DILI diagnosis	-6 *
**CMV**	
• Missing IgM anti-CMV and CMV-PCR data	0
• Negative (both IgM anti-CMV and CMV-PCR or at least one negative and one not done)	0
• Positive IgM anti-CMV or CMV-PCR	-2

• Positive IgM anti-CMV and CMV-PCR	-6
**EBV (IgM can be any IgM anti-EBV antibody, heterophilic test, monospot or EBV early antigen)**	
• Missing IgM and EBV-PCR data	0
• Negative (both IgM and EBV-PCR or at least one negative and one not done)	0
• Positive IgM or EBV-PCR	-2
• Positive IgM and EBV-PCR	-6
**HSV**	
• Missing IgM anti-HSV and HSV-PCR data	0
• Negative (both IgM anti-HSV and HSV-PCR or at least one negative and one not done)	0
• Positive IgM anti-HSV or HSV-PCR	-2
• Positive IgM anti-HSV and HSV-PCR	-6
**Drug reaction with eosinophilia and systemic symptoms (DRESS) or Steven Johnsons Syndrome (SJS)**	
• Absent or no information	0
• Present	1

Item 3 (risk factors) and 4 (concomitant drugs) from the original RUCAM were removed.

[1] R value is defined as ALT/ULN)/(ALP/ULN, with R ≥ 5 defining a hepatocellular, R ≤ 2 a cholestatic and 2 < R < 5 a mixed type injury.

[2] Agents with estimated half-life or pharmacodynamic effect ≥ 15 days.

[3] LiverTox categories: A: Well-known, well described, and characteristic signature with > 50 well reported cases in the literature; B: Known or highly likely to cause DILI with characteristic signature with 12-49 cases in the literature; C: Probably causes DILI. No characteristic signature with < 12 cases in the literature; D: Possible cause of DILI with < 3 cases in the literature. E: Unlikely to causes DILI due to extensive use. Cases in the literature may exist but are unconvincing. E*: Unproven but suspected to cause DILI. Suggestion of liver injury exists outside of published literature (e.g., trial data reported to regulatory agencies) X: Unknown. Agents recently approved or rarely used.

* The data suggests an alternative reason for DILI and that DILI is not explanatory of liver injury. The user should consider these cases as excluded or unlikely DILI with a total score of -6. If the user chooses to proceed, 6 points will be deducted from the current score and the user should recognize that DILI as the cause of liver injury is questionable due to inconsistent latency, dechallenge and this regardless of the total score obtained.

Abbreviations: AIH: Autoimmune hepatitis; ALT: Alanine aminotransferase; ALP: Alkaline phosphatase; ANA: anti-nuclear antibody; anti-Hbc: Anti-Hepatitis B core antibody; ASMA: anti-smooth muscle antibody; AST: Aspartate aminotransferase; CMV: Cytomegaly virus; DILI: Drug-induced liver injury; EBV: Epstein-Barr virus; IgG: Immunoglobulin G; IgM: Immunoglobulin M; HAV:

Hepatitis A virus; HbsAg: Hepatitis B surface antigen; HBV: Hepatitis B virus; HCV: Hepatitis C virus; HSV: Herpes simplex virus; PCR: Polymerase chain reaction; RNA: ribonucleic acid; RUCAM: Roussel Uclaf Causality Assessment Method; SIRS: Systemic inflammatory response; SD: Standard drink; TBIL: Total bilirubin; ULN: Upper limit of normal.
